# Self-Compassion and Psycho-Physiological Recovery From Recalled Sport Failure

**DOI:** 10.3389/fpsyg.2019.01564

**Published:** 2019-07-05

**Authors:** Laura A. Ceccarelli, Ryan J. Giuliano, Cheryl M. Glazebrook, Shaelyn M. Strachan

**Affiliations:** ^1^Department of Psychology, University of Manitoba, Winnipeg, MB, Canada; ^2^Faculty of Kinesiology and Recreation Management, University of Manitoba, Winnipeg, MB, Canada

**Keywords:** self-compassion, heart rate variability, sport, recovery, athletes, performance failure

## Abstract

Failure inherent to high-performance sport can precipitate emotional distress that can impair athletes’ performance and physical and mental health. Identifying factors that allow athletes to manage failure to sustain their health is critical. Self-compassion, treating oneself kindly in response to failure, may help athletes manage failure; it buffers against negative affective psychological responses, yet athletes often fear self-compassion. It is unknown whether the benefits of self-compassion extend to athletes’ physiological responses to failure and whether fear of self-compassion has an influence on psychological and physiological responses to failure, beyond self-compassion. The purpose of this study was to examine the influence of self-compassion on athletes’ psychological and physiological responses when recalling a sport failure and determine if fear of self-compassion exerted unique effects, beyond self-compassion. Participants (*n* = 91; *M* age = 21) were university or national-level athletes. In this laboratory-based, observational study, athletes were connected to a multi-modal biofeedback system to measure physiological responding at baseline, during a stress induction (imagining a past performance failure), and during a recovery period. Physiological responding was assessed according to athletes’ high-frequency heart rate variability (HRV), indexing parasympathetic nervous system activity, during the stress induction and recovery phase. Next, to assess psychological reactivity, athletes completed a series of scales (behavioral reactions, thoughts, and emotions). Regression analyses revealed that self-compassion predicted athletes’ HRV reactivity to the stress induction (β = 0.30, *p* < 0.05). There was no relationship between self-compassion and HRV recovery. Further, self-compassion predicted adaptive behavioral reactions (β = 0.46, *p* < 0.01), and negatively predicted maladaptive thoughts (β = −0.34, *p* < 0.01) and negative affect (β = −0.39, *p* < 0.01). Fear of self-compassion explained additional variance in some maladaptive thoughts and behavioral reactions. Results suggest that self-compassion promotes adaptive physiological and psychological responses in athletes relative to a recalled sport failure and may have implications for performance enhancement, recovery and health outcomes. Further, addressing athletes’ fears of self-compassion may also be important in promoting optimal psychological recovery.

## Introduction

Failure is common among high-performance athletes who pursue challenging goals and must maintain high performance standards (Smith et al., 2006; Davis et al., 2007). These failures can be challenging for athletes to accept and cope with given the pressures they feel to perform well, combined with the significant investment of time and energy required to participate in elite sport. Further, athletes are harshly criticized when they fail to meet performance expectations, and often endure the consequences of failing to meet expectations (i.e., lost playing time; withdrawn financial support) (Davis and Sime, 2005; Bauman, 2016). Though failure is common for athletes, the criticism and consequences that athletes experience when they fail, combined with the pressures and expectations to be “mentally tough” in the face of challenges (Hammond et al., 2013; Bauman, 2016) make experiencing and coping with failure challenging for athletes. These failure experiences may contribute to poor mental health among athletes (Davis et al., 2007; Hammond et al., 2013; Mosewich et al., 2014). Indeed, many athletes report feeling a diminished sense of self and emotional distress following performance failure (Davis et al., 2007; Sagar et al., 2007; Sutherland et al., 2014) and these failures can precipitate depressive symptoms, anger and decreased vigor (Jones and Sheffield, 2008; Hammond et al., 2013).

The emotional distress that athletes report following failure often takes the form of self-criticism, self-blame, obsession and rumination (Mosewich et al., 2013; Ferguson et al., 2014). Many athletes believe this way of relating to themselves is necessary for success in elite sport and without it, they will become complacent and fail to reach their potential (Sutherland et al., 2014; Rodriguez and Ebbeck, 2015). However, researchers suggest this response to failure can actually be counter-productive; responding to failure with self-criticism and harsh self-punishment undermines self-regulation, emotional recovery, stress management and performance (Powers et al., 2009; Tenenbaum et al., 2013; Ferguson et al., 2015) and is positively associated with emotional reactivity, avoidance and fear of failure (Sagar et al., 2007; Powers et al., 2009). Further, this response pattern increases athletes’ vulnerability to psychological distress and psychopathology (Powers et al., 2009; Hammond et al., 2013; Tenenbaum et al., 2013). Frequent failure experiences combined with these self-critical and ruminative tendencies that athletes endorse in response to failure may contribute to athletes experiencing equal or greater instances of mental health concerns than the general population (Reardon and Factor, 2010; Hammond et al., 2013). Given that athletes’ mental health issues are often discounted, overlooked or go undetected, the estimates likely under-represent the true state of mental health problems among athletes (Reardon and Factor, 2010).

There may also be physiological costs when athletes are self-critical about their failures. The autonomic nervous system plays an integral role in responding and adapting to changing stimuli in the environment. While the sympathetic branch of the autonomic nervous system is implicated in “fight-or-flight” responses and active mobilization, the parasympathetic branch has been implicated in so-called “rest-and-digest” processes that promote long-term health and restoration of the body (Thayer and Sternberg, 2006; Porges, 2007; Thayer et al., 2012). The relative activity of these systems can depend upon whether or not we perceive our environment as safe, such that parasympathetic activity is notably increased for organisms who perceive their environment to be safe (Thayer and Lane, 2000; Porges, 2007). The parasympathetic nervous system can be measured via high-frequency heart rate variability (HRV), the variation in beat-to-beat intervals of the heart at relatively short cycles (0.15 to 0.40 Hz in adults; Porges, 2007). Commonly referred to as respiratory sinus arrhythmia, HRV has been utilized for the assessment of parasympathetic activity at rest and in response to environmental challenges, with the parasympathetic branch acting to inhibit or slow heart rate during moments of regulation. When stressed, the parasympathetic branch releases its inhibitory influences on the heart, decreasing HRV and accelerating heart rate, as the sympathetic nervous system drives an excitatory and “locked in” or inflexible state (Porges, 2007; Thayer and Lane, 2009; Thayer et al., 2012). This inflexible state is typically less adaptable in terms of range of behaviors than when HRV is high. Such states characterized by low HRV are commonly reported in association with emotional arousal or dysregulation, negativity bias (the tendency to be overly attentive to negative or threatening stimuli) and an increased likelihood of disease and mortality (Thayer and Sternberg, 2006; Porges, 2007; Thayer and Lane, 2009). Sustained activation of the body’s stress response (i.e., persisting low HRV) can be brought on by ruminative thinking, obsession or self-criticism (McEwen and Wingfield, 2003; Gilbert, 2014), which can be detrimental to physical and mental health and can undermine performance (e.g., inhibits coordination, decision making, response time, and automatic skill execution; Davis and Sime, 2005; Bertollo et al., 2013; Tenenbaum et al., 2013). Thus, by examining a physiological marker of the parasympathetic nervous system, we can gain an understanding of the body’s state of responsiveness to environmental demands and well-being.

Performance failures cause significant distress for high-performance athletes (Davis and Sime, 2005; Hammond et al., 2013; Bauman, 2016). The stress that athletes feel when they encounter failure is compounded by their tendencies to respond to failure with harsh self-criticism, judgment, and rumination, which are psychologically and physically costly (Juster et al., 2010; Gilbert, 2014). Thus, in order to optimize the health and performance of athletes it is important for researchers and practitioners to identify factors that effectively regulate psychological and physiological reactions to stressful experiences, such as sport performance failure (Juster et al., 2010; Dupee et al., 2015). Self-compassion may represent one such factor. Self-compassion involves treating oneself with care and concern in times of struggle and consists of three integrated components (Neff, 2003a). The first, self-kindness versus self-judgment, involves alleviating one’s own suffering through self-care and concern versus harsh self-criticism. Next, mindfulness versus over identification, entails an open and balanced view of one’s emotions without avoiding them or over-identifying with them. Finally, common humanity versus isolation, is the acceptance that failure is a shared human experience rather than an isolated experience (Neff, 2003a).

Researchers theorize that self-compassion can provide people with the emotional safety in times of failure that allows them to see their shortcomings in an open and balanced way, without feeling threatened, or the need to avoid difficult emotions as a means of coping (Neff et al., 2005; Allen and Leary, 2010). Indeed, in the face of set-backs self-compassion is positively associated with individuals’ accurate self-appraisals and low levels of avoidance, negative affect and rumination (Neff et al., 2005; Leary et al., 2007; Breines and Chen, 2012). Further, self-compassion is positively associated with personal initiative and taking an approach (vs. avoidance) orientation to problems (Neff et al., 2005, 2007; Zhang and Chen, 2016). There is experimental evidence that inducing a self-compassionate state leads people to take more responsibility for their role in negative events, to view shortcomings as changeable and to be motivated to change when compared to control participants (Breines and Chen, 2012; Zhang and Chen, 2016). Thus, it is not surprising that self-compassion is positively related to emotional coping skills, and ability to repair negative emotional states (Neff, 2003b, Neff et al., 2005; Arimitsu and Hofmann, 2015).

In addition to the psychological benefits of self-compassion (Barnard and Curry, 2011), self-compassion may promote adaptive physiological regulation in response to failure. When people respond to failure with self-criticism, they may activate similar affect pathways, in this case a threat-defense system, as when they are being attacked by another person or experience a threatening event (Gilbert and Irons, 2005; Gilbert et al., 2006; Gilbert, 2014). This activation of the stress response can occur because the brain and nervous system respond similarly to internally generated images as to external stimuli (Gilbert, 2014). Sustaining this activation of the body’s threat response can increase one’s vulnerability to developing psychopathology and illness (McEwen and Wingfield, 2003; Thayer and Sternberg, 2006; Juster et al., 2010; Karatsoreos and McEwen, 2011; Gilbert, 2014). Responding to failures with self-compassion, rather than self-criticism, appears to encourage adaptive physiological processes, in particular increases in parasympathetic nervous system activity as indexed by high-frequency HRV (Rockliff et al., 2008; Arch et al., 2014; Svendsen et al., 2016). Both dispositional (Breines et al., 2015) and increased (Arch et al., 2014) levels of self-compassion are associated with adaptive autonomic reactivity following a laboratory acute stressor, seen as reduced sympathetic and increased parasympathetic nervous system activity. Relatedly, experimentally inducing a compassionate state using compassion-focused imagery increases participants’ HRV, suggesting that self-compassion could stimulate a soothing affect system in the body via the parasympathetic nervous system (Rockliff et al., 2008). The impact of compassion on elevated parasympathetic activity has been replicated multiple times, including four studies demonstrating that a compassion induction elevates participants’ HRV relative to a variety of control group contexts (Stellar et al., 2015).

Self-compassion may support well-being through promoting both adaptive psychological and physiological responses to failure that could be helpful to athletes as they cope with failure in the context of competitive sport. Researchers have examined the psychological benefits of self-compassion among female athletes and found that self-compassion negatively associated with guilt and shame, body consciousness, fear of failure, fear of negative evaluation (Mosewich et al., 2011) and predicted favorable performance evaluations (Killham et al., 2017). Not surprisingly, self-compassion positively related to psychological well-being, positivity, initiative and perseverance and negatively related to passivity, anxiety, negative affect and avoidance coping in response to emotionally challenging sport scenarios among female collegiate athletes (Ferguson et al., 2014, 2015; Reis et al., 2015). Researchers provide experimental support that self-compassion can promote healthy psychological responses to failure; a self-compassion intervention reduced female athletes’ self-criticism, rumination and concern over mistakes, and these results were maintained at a 4-week follow-up (Mosewich et al., 2013). While there is emerging support that self-compassion can promote adaptive psychological responses to failure, whether self-compassion offers *physiological* benefits to athletes has not been explored.

Despite the benefits of self-compassion in sport, athletes remain hesitant to adopt this approach, as doing so would be contradictory to the (supposed) formula for success of mental toughness and self-criticism when they fail (Sutherland et al., 2014; Rodriguez and Ebbeck, 2015). Indeed, the mindset that self-criticism is necessary for success has persisted in competitive sport (Sutherland et al., 2014). Moreover, athletes are expected to be “mentally tough” when they fail and failing to do so would be considered a sign of weakness (Reardon and Factor, 2010; Bauman, 2016). These prevalent beliefs and expectations make athletes fearful that being honest about the emotional distress that they feel when they fail and being gentler with themselves would lead to stigmatization or being seen as incapable (Sagar et al., 2007; Reardon and Factor, 2010; Hammond et al., 2013; Bauman, 2016). That is to say that athletes may not only show low levels of self-compassion, but they may actively *resist* or fear adopting a self-compassionate perspective. When attempting to implement Compassion-Focused Therapy among individuals with high self-criticism, Gilbert and Procter (2006) discerned that when trying to offer oneself compassion, many people are met with resistance and fear. Gilbert et al. (2011) defined this construct as fear of self-compassion, or the experience of difficulty or unpleasantness when extending kindness and understanding to oneself during times of distress (e.g., when we make a mistake or things go wrong in life). People high in fear of self-compassion experience self-compassion as threatening and actively resist this experience (Gilbert and Procter, 2006; Gilbert et al., 2011). Fear of self-compassion is a related but distinct construct from self-compassion (Gilbert et al., 2011; Joeng and Turner, 2015; Kelly et al., 2013) that positively associates with maladaptive psychological characteristics (i.e., feelings of inadequacy, self-hatred and self-criticism), a threat-defense response to compassionate experiences (i.e., low HRV and high cortisol; Rockliff et al., 2008), and impaired mental health (Gilbert et al., 2011; Joeng and Turner, 2015).

Fear of self-compassion is relevant to athletes given that it is amplified in highly competitive and evaluative environments, such as competitive sport (Gilbert et al., 2011; Mosewich et al., 2011; Gilbert, 2014). Competitive environments emphasize dynamics of inferiority and superiority, where individuals feel a need to be accepted and attain and sustain dominance which amplifies fears of subordination and exclusion (Gilbert, 2014). Indeed, people fear that by adopting self-compassion, they will become weak, lose their self-criticism and their standards will drop (Gilbert et al., 2011). Self-reported findings from athletes mirror these concerns and suggest they are fearful of adopting self-compassion because they will become complacent or be viewed negatively by others (Mosewich et al., 2014; Sutherland et al., 2014), despite the negative psychological consequences that are associated with this resistance in times of failure (Ferguson et al., 2015). Research examining the role of fear of self-compassion among athletes remains limited to a few studies, and researchers need to replicate and more fully understand the role of fear of self-compassion as it relates to self-compassion in sport. For example, whether fear of self-compassion acts as a barrier to effective psychological and physiological regulation beyond the effects of self-compassion is unknown.

Our primary purpose in the present study was to explore previously supported associations between self-compassion and psychological responses to failure among athletes and to provide a preliminary exploration of associations between self-compassion and physiological responses to failure. A secondary purpose was to determine if fear of self-compassion accounted for any additional variance in study outcomes beyond the effects of self-compassion.

## Materials and Methods

### Design and Participants

We conducted a power analysis using G^∗^Power (Erdfelder et al., 1996), based on an alpha level of 0.05, a power level of 0.95, and an effect size of 0.20 and determined that we required sample size of 90 participants. A total of 91 athletes completed this laboratory-based, observational study. Eligibility criteria included being currently selected to compete in their sport at a university or national level, free from psychological or physical conditions or medications that may alter their stress response, and the ability to recall a recent distressing sport failure or setback that they remembered well. Eligibility was completed online by 142 people; 24 were ineligible. From the 118 remaining eligible people, 27 did not complete baseline measures, stopped responding, or were unavailable for the laboratory session. Participants who completed the study and were included in the analyses were 91 adult athletes (*M* age = 21.4; *SD* = 3.47; range: 18 to 40) who were primarily single (94.4%), Caucasian (76.9%), university students (92%), with slightly more participation from females (58%) and represented a variety of sports with the most participation from track and field athletes (20.9%). Participants had spent an average of 4.19 years competing at their current level in sport (*SD* = 4.15) and were highly involved in their sport (*M* weekly training hours in competitive season = 15; *SD* = 5.78; *M* weekly training hours in the off-season = 9; *SD* = 5.43). A summary of participant characteristics can be found in Table 1.

**Table 1:** Participant characteristics.

Characteristic	N	%
**Ethnicity**		
Caucasian	70	77
African	5	6
Aboriginal	4	4
Asian	2	2
Latin American	1	1
East Indian	1	1
Philippine	1	1
Other	7	8
**Marital Status**	85	93
Single	3	3
Common Law	2	2
Other		
**Sport Type**		
Track and Field	20	22
Volleyball	18	20
Hockey	17	19
Soccer	8	9
Football	6	7
Basketball	4	4
Swimming	3	3
Cross Country	3	3
Rowing	3	3
Racquetball	2	2
Badminton	2	2
Ringette	2	2
Curling	1	1
Figure Skating	1	1
Rugby	1	1
**Gender**		
Female	53	58
Male	38	42

### Baseline Measures

#### Demographics

Participants reported their age, gender, marital status, current sport, sport history, year in sport at a university or national level and university major.

#### Self-Compassion

Self-compassion was assessed using the 26-item Self-Compassion Scale (Neff, 2003b). Participants responded on a five-point Likert scale ranging from 1 (*almost never*) to 5 (*almost always*). Six subscales assess the three facets of self-compassion and their opposing facets: mindfulness (over-identification), self-kindness (self-criticism) and common humanity (isolation). Negatively worded items were reverse scored. Means of each subscale were created and combined to create a grand self-compassion mean (Neff, 2003b). Higher scores on this scale indicate higher levels of self-compassion. The Self-Compassion Scale has good test-retest reliability, discriminant and concurrent validity and good internal consistency reliability (α = 0.92) and scale items have been found to be reliable among athletic samples (α = 0.87; Mosewich et al., 2011) including the present sample (α = 0.91).

#### Fear of Self-Compassion

Fear of self-compassion was assessed using the 15-item Fear of Self-Compassion Scale (Gilbert et al., 2011); participants rated their agreement with statements on a five-point scale: 0 (*don’t agree at all)* to 4 (*completely agree*). Items were summed to represent and overall score. Higher scores indicate higher levels of the construct. Items of the Fear of Self-Compassion Scale show good internal consistency (α = 0.85, 0.95; Gilbert et al., 2011; Kelly et al., 2013), including within the present sample (α = 0.89) and the scale has been used previously with athletic samples (Ferguson et al., 2015).

#### Imagery Ability

Given that imagery ability may impact participants’ reactivity during the stress induction (Kwekkeboom, 2000), imagery ability was included as a possible control variable in this study. The Motivational General-Arousal (MG-A) subscale of the Motivational Imagery Ability Measure for Sport (MIAMS), was chosen in order to assess participant’s ability to generate emotional experiences associated with sport (e.g., anxiety) using imagery (Gregg and Hall, 2006). This subscale assesses participants ease of forming the image, and intensity of the emotional experience generated by the image. To complete this scale, participants were asked to generate images associated with four different sport scenarios (e.g., feeling anxious before a sporting competition), and rate the ease of forming the image (four-items) and the emotional experience (four-items) created by the image on scale from 1 (*no emotion)* to 7 (*very strong emotion).* Emotion and ease were assessed separately (Gregg and Hall, 2006). The MG-A subscale of the MIAMS has shown acceptable reliability (α = 0.74 emotion; α = 0.73 ease), including within the present sample (α = 0.70 emotion; α = 0.69 ease) among athletic samples.

#### Self-Esteem

The 10-item Rosenberg self-esteem scale (RSES; Rosenberg, 1965) assessed self-esteem. Participants indicated the extent to which they agreed with each statement on a scale from 1 (*strongly disagree*) to 4 (*strongly agree*). Negatively worded items were reverse scored, and scores from all 10 items were summed. Higher scores represent higher levels of self-esteem. The scale shows good predictive, concurrent, construct validity and scale items were internally consistent in other (α = 0.87; Rosenberg, 1965) and in the present sample (α = 0.82). The scale shows acceptable psychometric properties when used with athletic samples (e.g., Mosewich et al., 2011) and has been used previously as a control variable alongside self-compassion (Mosewich et al., 2011).

### Laboratory Measures

#### High-Frequency Heart Rate Variability

Participants’ HRV was assessed in the frequency-domain according to the natural log of the total power of the high-frequency band (0.15–40 Hz) (Task Force of The European Society of Cardiology and The North American Society of Pacing and Electrophysiology, 1996) using a ProComp Infiniti (Thought Technology, Montreal, QC, Canada) multi-modal biofeedback system. This system is suitable for assessing physiological markers such as high-frequency HRV (e.g., Shaw et al., 2012; Heathers et al., 2014). Data was assessed by measuring participant’s blood volume pulse using a photo-plethysmograph sensor on the palmer surface of the non-dominant index finger at a sampling rate of 2048 Hz (Combatalade, 2010; Shaw et al., 2012). This method is considered a reliable and valid method of assessing HRV in typically developing samples (Heathers et al., 2014). Recordings were utilized from three, 120-s phases: Before (baseline assessment), during (reactivity) and following stress induction (recovery). Recordings longer than 60 s have been demonstrated to show good reliability for assessing HRV in athletes (Esco and Flatt, 2014).

#### Emotional Difficulty

A single item had athletes rate how “emotionally difficult” the scenario was for them on a scale from 1 (*not at all)* to 6 (*extremely*) which has been used in past research with athletes (Ferguson et al., 2015; Reis et al., 2015). The item served as a manipulation check to ensure that the recalled sport scenarios were distressing for the athlete at the time that they occurred.

#### Image Quality

Athletes rated the extent to which the generated failure image was easy to generate, arousing, clear, meaningful, emotional, and useful (six-items) on a scale from 1 (*not at all easy to form)* to 7 (*very easy to form).* A mean score was computed from the sum of six items to assess overall image quality. This manipulation check ensured the effectiveness of the stress induction. This measure was developed based on recommendations from imagery researchers (see Lang, 1979; Gregg and Hall, 2006; Hammond et al., 2012).

### Assessment of Outcomes

#### Psychological Responses to Failure Scenarios

Participants failure-related behavioral reactions, thoughts and emotions were assessed as an indicator of athletes’ psychological reactivity. While there are no psychometric properties for these measures, they have been used in studies conducted among university students (Leary et al., 2007) and athletes (Reis et al., 2015).

##### Behavioral reactions

Using a scale range of 1 (*not at all*) to 6 (*extremely*), participants rated how much they reacted in each of nine ways (e.g., “I took steps to fix the problem or made plans to do so”) at the time of the sport failure.

##### Thoughts

Participants rated the extent to which each of the six thoughts about the failure scenario were relevant for them on a scale ranging from 1 (*I did not think this thought at all*) to 5 (*I kept thinking about this thought*). All individual thought items were analyzed as is consistent with past research (see Leary et al., 2007; Reis et al., 2015).

##### Emotions

Participants rated the extent to which they felt 16 emotions on a scale from 1 (*not at all*) to 6 (*extremely*), at the time that the failure took place. The 16 terms were divided into four subscales: sad (four items: sad, dejected, down, and depressed), anxious (four items: nervous, worried, anxious, and fearful), angry (four items: irritated, angry, hostile, and mad), and self-conscious (four items: embarrassed, humiliated, guilty, and ashamed). Scores from individual terms within each of the four subscales (i.e., sadness, anxiety, anger, and self-conscious emotions) were summed to create subscale scores, (Leary et al., 2007; Reis et al., 2015).

#### Physiological Reactivity

Reactivity was assessed according to recommendations (Laborde et al., 2018) to compute a difference score (i.e., stress induction – baseline) to quantify changes in participants’ mean value of HRV during the stress induction (120-s) relative to participant’s individual baseline values (120-s).

#### Physiological Recovery

Following recommendations (Laborde et al., 2018), the mean value of participants’ HRV during recovery (120-s) was subtracted from their mean value of HRV during the stress induction (120-s).

### Procedures

#### Recruitment, Eligibility and Baseline Assessment

Upon attaining institutional ethics approval, we recruited participants from two Canadian universities and a national sport center, through requests to teams, posters, and word of mouth. We emailed interested participants the online eligibility survey. Eligible participants provided informed consent and completed the baseline survey online which included measures of self-compassion, self-esteem, fear of self-compassion and demographics.

#### Laboratory Session

Two days prior to a scheduled laboratory session, participants were briefed regarding pre-laboratory session eligibility criteria. Participants were asked to ensure that they were free from substances (e.g., alcohol, drugs, or medications) or physical conditions (e.g., concussion, lack of/poor sleep, or illness) that may impact their body’s physical responses to stress (Svendsen et al., 2016; Laborde et al., 2017), in order to ensure that their physiological data was as reliable as possible. Participants were asked to reschedule their session if they did not meet the pre-laboratory session criteria. The first author conducted sessions at laboratories at the universities and the sports center. After a brief orientation to study procedures, the researcher connected the participant to the equipment used to record physiological responses (ProComp Infiniti, Thought Technology, Montreal, QC, Canada).

The researcher then instructed the participant to remain calm and relaxed for a 2-min acclimation period, followed by a 2-min baseline assessment of physiology. A 2-min acclimation period is considered acceptable in order to alleviate participants’ anxieties or nerves and to help them to feel comfortable with being connected to the physiological recording equipment (Heathers et al., 2014; Laborde et al., 2017). The instructions for the acclimation and baseline assessments were to sit comfortably and relaxed with both of their feet flat on the floor, hands on their thighs and palms facing up (Laborde et al., 2017). Participants were told to remain as still as possible, as movement may interfere with the recording. Next, participants underwent a stress induction. To induce a stress response, participants were asked to imagine a recent sport failure or setback with their eyes closed, for 2 min, using a guided imagery script read aloud by the researcher. Participants were instructed to remain seated with their eyes closed and provided the same instructions that were delivered during the baseline recording (feet flat on the floor, hands on their thighs and palms facing up) and were again instructed to remain as still as possible. The imagery script was read aloud to prompt participants to image their past failure for the entire 2-min period. Prompts were provided followed by brief pauses to allow participants to generate their images. The imagery script provided prompts to promote elaboration of participant’s images and the emotion associated with their failure experience. Further, providing ongoing prompts helped to ensure that participants were thinking about the failure scenario for the duration of the stress induction. The imagery script was developed based on imagery best practice (Lang, 1979; Hammond et al., 2012) and by consulting with a sports imagery expert. The imagery script was pilot tested prior to commencing data collection to ensure it extended for the duration of the 2-min time frame. Moreover, the same researcher (the first author) read and followed the rehearsed script for all participants to provide uniform delivery. The imagery script for the stress induction was as follows:

Remember a time when you failed… Maybe you made a costly mistake, failed to meet an important goal, or experienced a setback in your sport progress…... Imagine this experience……. In your mind, really try to take yourself back to this experience…… Remember your expectations leading up to this… Remember the pressures that you felt… Imagine what you were looking forward to and your hopes… Then remember the situation unfolding as it did… Remember where you were, what your surroundings looked like, who was there……. …...Take yourself back to the stressful situation in as much detail as possible……. …….. Really focus on the feelings that you had…... Disappointment, anger, frustration, despair… Try to remember those feelings in as much detail as possible…... Really allow yourself to feel them… Remember the changes in your body……tension, anxiousness, uneasiness……. Imagine this scenario in as much detail as possible…… Even after this moment or situation had passed, notice any feelings that remain: tension, regret, uneasiness......... Really try to take yourself back to the feelings and emotions that you experienced……... Now, please take a deep breath and gently open your eyes.

The stress induction was followed by a 2-min recovery period where participants were instructed to relax their body and their mind with their eyes open. The researcher then disconnected the participant from the equipment and the participant completed the psychological laboratory measures (see section “Laboratory Measures” in “Materials and Methods”). The researcher then debriefed participants by providing a detailed explanation of the study’s purposes and how they could access the results and then thanked the participant for their time.

## Results

### Data Management and Preliminary Analyses

Artifacts from physiological data were removed using visual inspection and manually corrected to ensure accurate placement of individual heart beats (Combatalade, 2010). We followed recommendations for cleaning and preparing the physiological and psychological data (Pallant, 2007; Tabachnick and Fidell, 2013).

Correlational analyses (Pearson product moment correlations) showed that main study outcomes were correlated in the expected directions. Self-compassion was negatively associated with fear of self-compassion and self-esteem and fear of self-compassion was associated with self-esteem (see Table 2).

**Table 2:** Descriptive statistics and correlations of main variables

Measure	1	2	3	4	5	*M*	*SD*	α
(1) Self-compassion	–					3.10	0.56	0.91
(2) Fear of self-compassion	−0.48^∗∗^	–				17.91	9.49	0.89
(3) Self-esteem	−0.61^∗∗^	0.50^∗∗^	–			19.80	4.44	0.82

*^∗^p < 0.05, ^∗∗^p < 0.01.*

We considered covariates used in past research: age, gender; (Corrales et al., 2012) and imagery ability (Kwekkeboom, 2000; Gregg and Hall, 2006). We also considered emotional difficulty of the recalled failure and time since the failure as covariates given the possibility that they may influence study outcomes. We included these variables as covariates if they were correlated with the outcome variable (Tabachnick and Fidell, 2013) as determined by Pearson product moment correlations. When we included the aforementioned variables in our analyses, none had an effect on our outcome variables. Finally, given the associations between self-esteem and self-compassion, and the past precedent and recommendation to control for self-esteem when assessing self-compassion (Neff, 2003a), self-esteem was automatically included as a covariate in all analyses.

Note that all analyses reported herein were replicated with respiration rate included as a covariate. In all cases, respiration rate had no effect on the pattern of results reported. Thus, we excluded respiration rate from analyses reported here.

### Recalled Performance Failures

Repeated measures analysis of variance (ANOVA) analyses of high-frequency HRV were conducted across three timepoints (baseline, stressor and recovery) to ensure that our stress induction produced the expected changes in participants’ HRV. This analysis revealed a main effect of Time, *F*(1, 90) = 5.30, *p* < 0.05). A pairwise comparison revealed that the stress induction (failure recollection) produced significant reductions in HRV from baseline to stressor (*p* < 0.001) in the expected direction (*M* decrease = 39%), which is consistent with a stress response. Moreover, additional pairwise comparisons showed that the differences in HRV approached the conventional level of significance between baseline and recovery (*p* = 0.07) and stressor to recovery (*p* = 0.07). The reduction in HRV during a stressor is consistent with differences observed in other studies that have utilized a standard laboratory stressor (Arch et al., 2014; Wawrzyniak et al., 2016) and greater than reductions observed during personally relevant, stressful imagery task (Levine et al., 2016). Participants reported that they were able to easily generate their failure images (*M* = 5.26 out of 7, *SD* = 1.24), and these images were clear (*M* = 5.63 out of 7, *SD* = 1.17), emotional (*M* = 4.60 out of 7, *SD* = 0.99) and meaningful (*M* = 5.20 out of 7, *SD* = 1.10). Further, participants reported that during the imagery task, they felt the emotions of the image (*M* = 4.70 out of 7, *SD* = 1.10) and used the image (*M* = 5.25 out of 7, *SD* = 0.96) for the duration of the task. Our participants showed slightly higher ease of imaging compared to participants in a study by Williams et al. (2017); *M* = 4.05 out of 7 in stress condition who used a similar scale to assess participant’s ease of imaging a stressful imaging task. Consideration of participants’ physiological and self-reported responses suggest that the imagery induction successfully induced a stress response in the expected directions.

### Main Analyses

**FIGURE 1:**
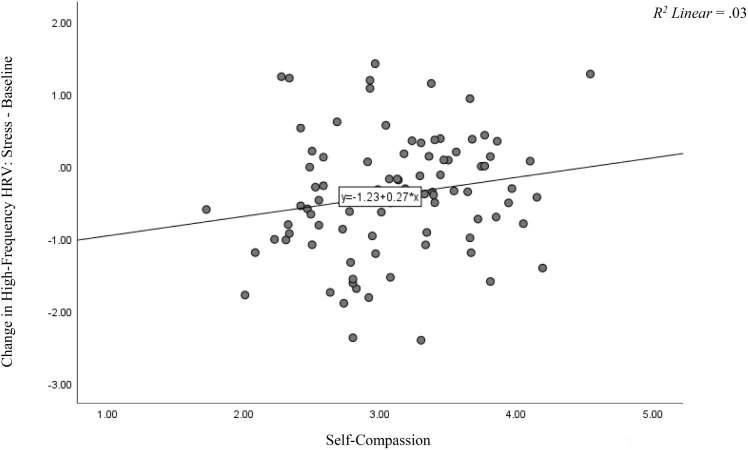
Difference Score of High-Frequency HRV for Stressor – Baseline and Self-Compassion. This figure depicts the change scores in participants’ high-frequency HRV from the stress induction subtracted from their baseline scores relative to their self-compassion scores. Self-compassion was positively related to parasympathetic nervous system activity (high-frequency HRV) during the stressor (*p* = 0.02).

Hierarchical linear regression analyses were conducted in order to test three research questions. Self-esteem was included as a covariate in Step 1 of all analyses. The first hypothesis was partially supported; self-compassion was positively related to participants’ HRV reactivity during the stress induction, but it was not related to HRV during the recovery phase. The addition of self-compassion in Step 2 accounted for an additional 5.7% variance in participants’ HRV reactivity during the stress induction [*F*(1, 88) = 8.18, *R*^2^ = 0.06, *R*^2^ *change* = 0.06, *p* < 0.05], beyond the effects of self-esteem. That is, high levels of self-compassion were positively associated with parasympathetic nervous system activity during the stressor (high-frequency HRV). The size of this effect was small (*f*^2^ = 0.06). Inspection of the beta values revealed that only self-compassion (*beta* = 0.30, *p* = 0.02) accounted for participants’ HRV during the stress induction. A visual depiction of these data is presented in Figure 1.

Hierarchical regression analyses were conducted for all psychological outcomes of interest (i.e., behavioral reactions, thoughts, and emotions), with self-esteem entered in Step 1, followed by the main variable, self-compassion in Step 2. For behavioral reactions, combining eight of the nine items produced acceptable reliability (α = 0.78) so we analyzed these items as a composite “behavioral equanimity” measure. Six thought items and four emotion subscales (sad, anxious, angry, and self-conscious) were entered separately as the outcome variables in a series of hierarchical regression analyses. Results supported our second hypothesis that self-compassion was associated with all three aspects of psychological reactivity (i.e., behavioral equanimity, maladaptive thoughts, and negative affect), in the expected directions (see Tables 2–4), beyond the effects of self-esteem. Self-compassion was related to behavioral equanimity [*F*(1, 88) = 15.1, *R*^2^ = 0.21, *R*^2^ *change* = 0.13, *p* < 0.01) and had unique effects on this outcome (*beta* = 0.46, *p* < 0.01), such that the effects of self-esteem were no longer significant (*beta* = 0.03, *p* = 0.82). This was considered to be a medium sized effect (*f*^2^ = 0.27). Self-compassion related to one adaptive thought (“This is no worse than what other people go through”) and negatively related to maladaptive thoughts and emotions (see Tables 3, 4). Of note, with some thought items (e.g., thinking “I’m a loser,” “my life is really screwed up” and “I have bigger problems than most people do”), self-compassion and self-esteem had opposite effects; self-esteem was positively associated while self-compassion was negatively associated with these maladaptive thoughts.

**Table 3:** Results from hierarchical regression analyses: thoughts.

Thought item		β	R^2^	Δ R^2^	*f*^2^
“I seem to have bigger problems than most people do”	Step 1		0.04		
	Self-esteem	0.20			
	Step 2		0.10^∗^	0.06^∗^	0.11
	Self-esteem	0.01			
	Self-compassion	−0.31^∗^			
“In comparison o other people, my life is really screwed up”	Step 1		0.10^∗∗^		
	Self-esteem	0.32^∗∗^			
	Step 2		0.17^∗∗^	0.07^∗∗^	0.20
	Self-esteem	11			
	Self-compassion	−0.34^∗∗^			
“Why do these things always happen to me?”	Step 1	−0.00	0.04		
	Self-esteem	0.19			
	Step 2		0.10^∗^	0.06^∗^	0.11
	Self-esteem	−0.00			
	Self-compassion	−0.31^∗^			
“Everyone has a bad day now and then”	Step 1		0.02		
	Self-esteem	−0.12			
	Step 2		0.04	0.02	
	Self-esteem	−0.01			
	Self-compassion	0.18			
“I’m a loser”	Step 1		0.13^∗∗^		
	Self-esteem	0.36^∗∗^			
	Step 2		0.24^∗∗^	0.11^∗∗^	0.32
	Self-esteem	0.11			
	Self-compassion	−0.43^∗∗^			
“This is no worse than what other people go through”	Step 1		0.01		
	Self-esteem	0.09			
	Step 2		0.05	0.04^∗^	
	Self-esteem	0.25			
	Self-compassion	0.26^∗^			

*^∗^p < 0.05, ^∗∗^p < 0.01.*

**Table 4:** Results from hierarchical regression analyses: emotions.

Emotion subscale		β	R^2^	Δ R^2^	*f*^2^
Anxious	Step 1		0.02		
	Self-esteem	0.15			
	Step 2		0.08^∗^	0.06^∗^	0.10
	Self-esteem	−0.04			
	Self-compassion	−0.31^∗^			
Angry	Step 1		0.02		
	Self-esteem	0.13			
	Step 2		0.09^∗^	0.07^∗^	0.10
	Self-esteem	−0.07			
	Self-compassion	−0.33^∗∗^			
Sad	Step 1		0.11^∗∗^		
	Self-esteem	0.33^∗∗^			
	Step 2		0.16^∗^	0.05^∗^	0.20
	Self-esteem	0.15			
	Self-compassion	−0.29^∗^			
Self-conscious	Step 1		0.03		
	Self-esteem	0.15			
	Step 2		0.12^∗∗^	0.09^∗∗^	0.14
	Self-esteem	−0.09			
	Self-compassion	−0.39^∗∗^			

*^∗^p < 0.05, ^∗∗^p < 0.01.*

Related to our third hypothesis, fear of self-compassion did not associate with participants’ physiological responses. However, the addition of fear of self-compassion in Step 3 accounted for unique variance in one of the six thought items, when controlling for self-esteem and self-compassion, and approached the conventional level of significance for two additional thought items and behavioral equanimity. For the item “Everyone has a bad day now and then,” the model that included fear of self-compassion accounted for an additional 4.7% of the variance in the outcome beyond self-esteem and self-compassion [*F*(1, 87) = 4.45, *R*^2^ = 0.08, *R*^2^ *change* = 0.05, *p* < 0.05). The size of this effect was small (*f*^2^ = 0.10). Inspection of the beta values showed that fear of self-compassion was negatively associated with this outcome (*beta* = −0.26, *p* = 0.04), and exerted the opposite effect of self-compassion (*beta* = 0.11, *p* = 0.42). Further, the model that included fear of self-compassion in Step 3 approached the conventional level of significance for the thought item “In comparison to other people, my life is really screwed up” [*F*(1, 87) = 3.10, *R*^2^ = 0.20, *R*^2^ *change* = 0.03, *beta* = 0.20, *p* = 0.08], and exerted the opposite effect from self-compassion (*beta* = −0.28, *p* = 0.03). Additionally, the model that included the addition of fear of self-compassion approached the conventional level of significance for behavioral equanimity (*beta* = −0.19, *p* = 0.09).

## Discussion

The primary purpose of the present study was to examine the relationship between self-compassion and athletes’ physiological and psychological responses to a recalled sport failure, and further, to determine if fear of self-compassion accounts for unique variance in these outcomes beyond self-compassion. Self-compassion related to participants’ physiological response to a sport failure in terms of their HRV during recollection of a previous failure: participants with greater self-compassion showed a more regulated autonomic profile as indexed by greater parasympathetic nervous system activity. Self-compassion also associated with athletes’ adaptive psychological reactions to their recalled sport failure. Fear of self-compassion did not account for any unique variance in physiological responses beyond self-compassion, but accounted for unique variance in some psychological responses, beyond self-compassion.

### Self-Compassion and Physiological Reactivity

Self-compassion related to dampened physiological reactivity, in the form of blunted HRV withdrawal, during the stress induction. Consistent with other studies showing that compassion is positively related to parasympathetic nervous system activity, self-compassion appeared to dampen athletes’ physiological responding to the stress induction and was associated with higher HRV during the reactivity measurement.

This finding is consistent with other research where HRV was influenced by self-compassion in laboratory settings (Arch et al., 2014; Breines et al., 2015) and related to responses that reflect self-compassion such as adaptive cognitive processing, emotional regulation and behavioral responses to changing demands (Thayer et al., 2009, 2012). We are the first, to our knowledge, to show this relationship among athletes recalling a sport failure and we have reason to suggest the possibility that because of this association, self-compassion may offer a physiological resource to athletes when they encounter stress. Rather than prevent the experience of stress, self-compassion may promote willingness to confront and soothe oneself during stressful times. In line with this explanation, self-compassion has been positively associated with the ability to accept, tolerate and experience negative emotions rather than avoid or suppress those feelings (Neff et al., 2005, 2007; Allen and Leary, 2010; Diedrich et al., 2014) and has been negatively associated with avoidance, thought suppression and rumination (Neff et al., 2005, 2007; Barnard and Curry, 2011), which can have deteriorating effects on our physiological systems (Gilbert, 2014). Responding to experiences of stress with self-compassion rather than harsh self-criticism and judgment appears to have a soothing effect on the affect system and creates an adaptive physiological profile during stress (Arch et al., 2014; Gilbert, 2014; Breines et al., 2015). Consistent with these findings, our results showed that self-compassion was associated with higher parasympathetic tone during a stressor.

Indeed, the regulation of HRV has implications for well-being (Porges, 2007; Thayer et al., 2012), health (Thayer and Sternberg, 2006) and performance (Wawrzyniak et al., 2016; Williams et al., 2016) that may be beneficial to athletes. For example, sustained low or dysregulated HRV predicts behavioral risk factors (inhibition and risk aversion; Porges, 2007; Thayer et al., 2012) and psychological risk factors (negativity bias and poor emotional regulation; Thayer and Lane, 2009; Thayer et al., 2012) for psychopathology and adverse health (e.g., glucose dysregulation, inflammation and disrupted hypothalamic-pituitary axis function; Thayer and Sternberg, 2006; Thayer and Lane, 2009; Juster et al., 2010; Thayer et al., 2012). Further, withdrawal of parasympathetic tone during a stressor is associated with longer reaction times and lowered accuracy (Wawrzyniak et al., 2016; Williams et al., 2016), both of which have implications for sport performance. Though we did not test this in this study, our findings suggest that adopting self-compassion should facilitate optimal health and performance for athletes when they encounter performance stressors, given its association with high HRV (Arch et al., 2014). Researchers should employ experimental designs in future research to test this possibility.

### Self-Compassion and Physiological Recovery

Self-compassion was not related to athletes’ physiological recovery, in the form of HRV during recovery from the stress induction relative to baseline levels. It is possible that recovery from the stress induction is a longer process than could be measured in a 2-min recovery period immediately following the stressor. Consistent with this, prior studies suggest that HRV recovery takes approximately five to 10 min, whether the stressor is psychological (Ulrich et al., 1991) or physical (Seiler et al., 2007). However, other researchers have shown that utilizing a recording time longer than 60 s of HRV data were reliable among collegiate athletes (Esco and Flatt, 2014). Additionally, the timeframe that we used (2-min stress induction and recovery periods) may better reflect the demands of many sport contexts, where athletes are required to rapidly respond to failures compared to longer recording periods of 5 min or more. As suggested below, future studies should examine a longer induction and recovery procedure, to unpack physiological dynamics related to reactivity and recovery from stress.

### Self-Compassion and Psychological Reactions

In our study, self-compassion was also positively associated with adaptive *psychological* reactions to a past performance failure or setback in terms of behavioral equanimity, as well as many indicators of adaptive thoughts and low negative affect. Our findings replicate those of other researchers examining self-compassion among athletes (Mosewich et al., 2013; Ferguson et al., 2015; Reis et al., 2015) and general samples (Leary et al., 2007; Arimitsu and Hofmann, 2015) who have shown that being self-compassionate protects against negative affect and promotes equanimous thoughts and actions. Researchers argue that, in the face of hard times, self-compassion promotes a balanced awareness of, and reduction in difficult emotions, a desire to soothe the self rather than ruminate about and over-identify with failure, connection with others, and motivation to think and behave in ways that sustain well-being (Neff, 2003a; Allen and Leary, 2010; Barnard and Curry, 2011; Terry and Leary, 2011). Ours’ and others’ findings (Mosewich et al., 2013; Ferguson et al., 2015; Reis et al., 2015) suggest that self-compassion is positively associated with this adaptive psychological responding to failure in athlete populations. However, relatively few studies have examined self-compassion among athletes and researchers need to continue this line of investigation in order to more fully understand the role of self-compassion when dealing with performance failure (e.g., prospective and experimental designs; examinations of mediators and moderators).

Results from our study support others that suggest that self-compassion is positively associated with adaptive psychological reactions to sport (Reis et al., 2015) and exercise failures (Semenchuk et al., 2018), beyond the effects of self-esteem. We found that for some items (e.g., thinking “I’m a loser,” “my life is really screwed up” and “I have bigger problems than most people do”), self-compassion and self-esteem had opposite effects; self-esteem was positively associated while self-compassion was negatively associated with these maladaptive thoughts. According to Neff (2003b), a drawback of self-esteem is that maintaining high self-esteem involves an increased reliance on showing/feeling superiority over others and meeting performance standards. Thus, Neff and others propose that the beneficial effects of self-esteem can break down when performance standards are not met Neff (2003b), for instance in the case of performance failure, and can lead to negative psychological outcomes (e.g., negative affect and displacing responsibility; Leary et al., 2007; Neff and Vonk, 2009). Self-compassion, alternatively, allows individuals to face and experience negative feelings associated with failure, and turn those feelings into positive experiences of kindness, learning and understanding, and it promotes acceptance of responsibility without dismissal, blame to others or harsh self-judgment (Neff, 2003b; Neff and Vonk, 2009). That is, self-compassion permits individuals to maintain positive feelings toward the self, even when performance standards are not met. Our results and other’s results are consistent with Neff (2003b) arguments that suggest targeting self-compassion may be a more useful approach than self-esteem when dealing with failure. Thus, while both self-esteem and self-compassion can have value for athletes, a growing body of research suggests that in some instances, self-compassion may be a more useful resource than self-esteem – and one of those instances may be when athletes must manage difficult experiences associated with sport such as failure.

### Fear of Self-Compassion

Despite the benefits associated with self-compassion for athletes (Mosewich et al., 2013; Ferguson et al., 2015; Reis et al., 2015), athletes are hesitant to adopt this approach (Ferguson et al., 2014; Sutherland et al., 2014) because they fear that being self-compassionate will lead to poor performance. Contrary to these other findings, we found that athletes are not overly fearful of self-compassion (*M* = 17.91; scale range = 0–60) compared to highly self-critical samples (Kelly et al., 2013; *M* = 32.85) and scored similarly to another athletic sample (Ferguson et al., 2015; *M* = 15.18). However, we did find that fear of self-compassion was positively associated with some negative psychological reactions (e.g., “In comparison to other people, my life is really screwed up”) and negatively associated with positive psychological thoughts (e.g., thinking “everyone has a bad day now and then”) and behavioral reactions to a sport failure beyond self-compassion. These results are consistent with other’s (e.g., Ferguson et al., 2015) and challenge athletes’ assertions that self-criticism is necessary for growth and improvement in sport (Ferguson et al., 2014; Rodriguez and Ebbeck, 2015). Moreover, fear of self-compassion did not explain any of the variance in physiological responding beyond self-compassion. Given that fear of self-compassion involves an active resistance to extending compassion toward the self (Gilbert et al., 2011), it may be that fear of self-compassion’s relationship with physiological, and possibly psychological, responses is more apparent when the opportunity to be self-compassionate is made salient (e.g., Rockliff et al., 2008), which was not the case in this study. Therefore, fear of self-compassion may be more relevant in an intervention or experimental induction where athletes are taught or urged to put self-compassion in place in response to a failure. Further investigation is needed in order to understand when and how fear of self-compassion is distinct and dominant relative to self-compassion.

### Strengths and Limitations

Our study had a number of strengths. First, we followed a well-controlled, laboratory scenario which is appropriate for early stages of research (Czajkowski et al., 2015). Further, the stress induction (imagery task) was informed by imagery best practice and developed in consultation with an expert in the field of sports imagery. The effectiveness of the stress induction was confirmed by physiological changes and additional self-reported manipulation checks.

A limitation of this study is the reliance on recalled stimuli, to ensure personal relevance, to induce changes in physiological state rather than an immediate stimulus (e.g., a novel laboratory stressor or a real-life failure situation). In the future, researchers should examine whether self-compassion associates with adaptive physiological responding to standardized laboratory stressors or, seek out practical ways of assessing responses to more recent failures than assessed presently. Additionally, due to our use of 2-min recording intervals, our results should be considered tentative. Although some researchers suggest that recording times as short as 1 min can be considered reliable when assessing HRV (Esco and Flatt, 2014; Laborde et al., 2017), future studies should utilize longer recording times in order to understand the time course of the effect that we observed. It is possible that a longer induction and recovery protocol would enable better identification of HRV reactivity and recovery dynamics resulting from the imagery task. It should also be noted that here we used photoplethysmography as a proxy for electrocardiogram activity, and although these two measures may diverge under conditions of acute stress (Schäfer and Vagedes, 2013), photoplethysmography has been demonstrated to reliably assess acute stress reactivity (Charlton et al., 2018). Future studies are needed to determine whether the stress reactivity effects reported here would be more pronounced in electrocardiogram-derived measures of HRV. Finally, although there are theoretical connections between HRV, self-compassion and performance, performance was not assessed directly. As such, the relationship between self-compassion and performance is still unclear and is an important direction for future research.

## Conclusion

We found physiological support, in the case of high frequency HRV, to complement existing self-reported findings that self-compassion promotes adaptive emotional regulation and psychological reactivity to failure and stress, among athletes. Athletes with higher levels of self-compassion showed adaptive psychological and physiological responses relative to a recalled sport failure compared to those lower in self-compassion. This is encouraging given that individual’s stress responses may be consistent and easily replicated across contexts and stimuli (Andreassi, 2007). It is promising that self-compassion emerged as a protective factor for athletes’ parasympathetic reactivity during a stressor, suggesting that athletes with more self-compassion are better able to maintain calming influences on their physiological state. However, given that this is the first study to show this relationship among athletes, our results are preliminary and should be interpreted with caution. More research should be conducted in order to replicate our findings and to more fully understand the relationship between self-compassion and physiological reactivity when dealing with stress. Nonetheless, these findings provide evidence that self-compassion is relevant and beneficial for athletes and offer additional support for the ability of self-compassion to impact physiological responding to stress. Based on our results we suggest that athletes can benefit from developing self-compassion, but care should also be taken to address athletes’ apprehension and resistance to adopting this approach when they fail.

## Data Availability

The datasets generated for this study are available on request to the corresponding author.

## Ethics Statement

This study was carried out in accordance with the recommendations of the “University of Manitoba Research Ethics and Compliance committee.” All subjects provided their informed consent online accordance with the Declaration of Helsinki. The protocol was approved by the “University of Manitoba Research Ethics and Compliance committee.”

## Author Contributions

LC and SS developed the design of the study. LC conducted the laboratory sessions and surveyed the data collection, organized the database and carried out the statistical analyses for the psychological variables, and wrote the first draft of the manuscript. LC, RG, and CG organized the physiological data and conducted the data analyses for the physiological variables. All authors contributed to the manuscript revision, and read and approved the submitted version.

## Conflict of Interest Statement

The authors declare that the research was conducted in the absence of any commercial or financial relationships that could be construed as a potential conflict of interest.
